# Efficacy and safety of artesunate for patients with IgA nephropathy: a study protocol for a multicenter, double-blind, randomized, placebo-controlled trial

**DOI:** 10.1186/s13063-022-06336-3

**Published:** 2022-05-25

**Authors:** Qi Chen, Zi Wang, Jicheng Lv, Lijun Liu, Hang Li, Weiwei Sun, Yanhong Huo, Yingbo Guo, Cun Shen, Shichao Li, Zhenjie Chen, Jingwei Zhou

**Affiliations:** 1 Department of Nephrology, Dongzhimen Hospital, The First Affiliated Hospital of Beijing University of Chinese Medicine, No. 5 Haiyuncang, Dongcheng District, Beijing, 100007 China; 2 Renal Division, Peking University First Hospital, Institute of Nephrology, Peking University and Key Laboratory of Renal Disease, Ministry of Health of China, Beijing, 100034 China; 3 Department of Nephrology, Peking Union Medical College Hospital, Beijing, 100005 China; 4 Department of Nephrology, the 7th Medical Center of PLA General Hospital, Beijing, 100700 China; 5 Department of Nephrology, Dongfang Hospital, The Second Affiliated Hospital of Beijing University of Chinese Medicine, Beijing, 100078 China; 6 Department of Nephrology, Beijing Chinese Medicine Hospital Affiliated to Capital Medical University, Beijing, 100010 China

**Keywords:** IgA nephropathy, Artesunate, Proteinuria

## Abstract

**Background:**

IgA nephropathy is the most common glomerular disease and is a common cause of progression to end-stage renal disease in patients with kidney diseases. Proteinuria levels are critical for the prognosis of patients with IgA nephropathy, but many patients are still unable to effectively control their proteinuria levels after receiving RAAS blockers. Antimalarial drugs have shown good efficacy in the treatment of kidney disease in previous studies; however, there have been no strictly designed randomized controlled trials to confirm the clinical efficacy of artesunate for treating IgA nephropathy patients. Therefore, we designed this clinical trial to compare the effect of artesunate versus placebo in patients with IgA nephropathy.

**Methods:**

This study is a randomized, double-blind, three-group-parallel, placebo-controlled clinical trial. One hundred and twenty eligible IgA nephropathy patients at risk of progression will be randomly divided into the artesunate 100-mg group, artesunate 50-mg group, and placebo group. Changes in proteinuria and renal function will be measured 6 months after the intervention. The levels of Gd-IgA1 and anti-Gd-IgA1 in the patient’s blood will also be tested to explore the possible immune mechanisms.

**Discussion:**

Clinical evidence supporting artesunate treatment of IgA nephropathy is currently lacking, and we expect that the results of this trial will provide high-quality clinical evidence for artesunate as a treatment option for IgA nephropathy in the future.

**Trial registration:**

Chinese Clinical Trial Registry ChiCTR2000038104. Registered on 10 September 2020

**Supplementary Information:**

The online version contains supplementary material available at 10.1186/s13063-022-06336-3.

## Background

Immunoglobulin A (IgA) nephropathy, characterized by prominent mesangial IgA deposition that is obfuscated on an immunofluorescence microscope, is the most common glomerular disease in the world [1]. The natural history of IgA nephropathy is highly heterogeneous; although the clinical data have shown that 50% of IgA nephropathy patients experience a sustained clinical remission, up to 40% of patients still develop end-stage renal disease (ESRD) within 20 years, and up to 40% experience a persistent decline in renal function [2–4]. The pathogenesis of IgA nephropathy is related to a “multi-hit” autoimmune process [5], which involves increased production of atypical galactose-deficient mucosal-type IgA1 antibodies, with the formation of anti-IgA1 autoantibodies, deposition of IgA1-containing immune complexes within the glomerular mesangium, and incitement of a nephritogenic inflammatory response by these immune complexes. Based on these underlying mechanisms, immunosuppressive drugs, including corticosteroids, are a potentially effective treatment option. However, the latest KDIGO Clinical Practice Guideline recommends that only patients who remain at high risk of progressive CKD (defined as proteinuria >0.75–1 g/day despite 90 days of optimized supportive care) should be considered for a 6-month course of glucocorticoid therapy, and the important risk of treatment-emergent toxicity must be discussed with patients (2B, moderate-quality evidence) [6]. This recommendation takes into account the current uncertainty over the safety and efficacy of existing immunosuppressive treatment choices [7] and the risk of serious adverse events (deaths related to infectious complications in the use of glucocorticoids [8], so for the patients who remain at high risk of progression in IgAN and eGFR ≥30 ml/min per 1.73 m^2^, the opportunity to take part in a clinical trial to search for safer and more effective alternatives to corticosteroids should be offered. Although the latest rituximab, eculizumab, and targeted-release budesonide may be potential treatments for IgA nephropathy, they are still far from being introduced into routine clinical practice because of their high cost and unclear risks of adverse events [9–11]. In the absence of more effective and available control therapies for IgA nephropathy with a high risk of progression, easier and safer supportive therapies are warranted.

Antimalarial drugs may provide a new treatment approach for the management of IgA nephropathy. In a recent randomized controlled study, IgA nephropathy patients with RAAS blockers received hydroxychloroquine (HCQ) for 6 months and had a nearly 50% reduction in proteinuria, which was significantly different from patients who received RAAS blockers without HCQ (a 10% increase) [12]. HCQ has also been reported to reduce the systemic lupus erythematosus disease activity index (SLE-DAI) and mortality in lupus nephritis and is currently the standard treatment for lupus nephritis [13]. The immunomodulatory and anti-inflammatory mechanisms of HCQ seem to interfere with lysosomal activity, inhibit antigen presentation and Toll-like receptor signaling at different levels, and thus affect T cell- and B cell-related immune inflammation, thereby reducing kidney injury and reducing proteinuria levels [14–16]. Importantly, these studies also identified fewer side effects with HCQ than with corticosteroids, which gives us more confidence that antimalarial drugs may be an effective management measure for IgA nephropathy.

Artesunate (ART) is a derivative of artemisinin (ARS), a component extracted from the traditional Chinese herb *Artemisia annua L.*, with improved pharmacological properties. ART has similar immunomodulatory effects to other antimalarial drugs [17], such as hydroxychloroquine (HCQ) and chloroquine (CQ), but has different active ingredients, which leads to the lack of risk of visual loss caused by HCQ [18]. Many experimental pieces of evidence have shown that ARS and its derivatives (ARSs) have good anti-inflammatory and immunomodulatory functions in SLE, rheumatoid arthritis, multiple sclerosis, inflammatory bowel disease, and other immune diseases [19]. ARS has been shown to improve podocyte injury, improve podoid fusion, and upregulate the expression of Nephrin and Podocin proteins in the podocytes of adriamycin nephropathy mice [20], and SM934 (an ARR) can reduce proteinuria, circulating antibodies, immune complex deposition in tissues, and podocyte injury and finally delay the progression of membranous nephropathy by inhibiting TGF-β1 expression and reducing the Smad2/3 expression pathway in Heymann nephritis mice [21]. More importantly, there is experimental evidence that ART combined with HCQ can effectively reduce 24-h urinary protein, IgA, and IgG immune complex deposition in the glomeruli of IgAN rats to reduce kidney damage through the NF-κB/NLRP3 signaling pathway [22]. Therefore, we hypothesized that ART might have the same excellent effects as HCQ in alleviating kidney injury and reducing proteinuria by inhibiting the activation and proliferation of B cells, inhibiting the generation of Gd-IgA1, and inhibiting inflammatory cytokines.

However, although a large amount of experimental evidence has been accumulated, there is still a lack of randomized controlled clinical trials to verify the potential effect of ART in the treatment of IgA nephropathy. This multicenter, double-blind, randomized, placebo-controlled study was designed to estimate the efficacy and safety of ART in IgA nephropathy. We present this protocol, which was prepared in accordance with the SPIRIT reporting checklist.

## Methods and design

### Study design

This study is designed as a double-blind, randomized, three-group-parallel, placebo-controlled superiority trial. One hundred and twenty patients with IgA nephropathy will be recruited, and the overall study design will be divided into two phases. In the pre-randomization phase, all participants will be confirmed to have received at least 3 months of RAAS blocker therapy to ensure that the patients have reached optimal blood pressure control (130/80 mmHg, the ideal range specified by the KDIGO2012 clinical practice guidelines) or have received the maximum tolerated dose of ACE inhibitor or ARB before entering the next phase. After qualification confirmation and informed consent, the enrolled patients will enter the post-randomization intervention phase. All participants will be randomly divided into the ART 100-mg group, ART 50-mg group, and placebo group for 6 months of intervention.

**Fig. 1:**
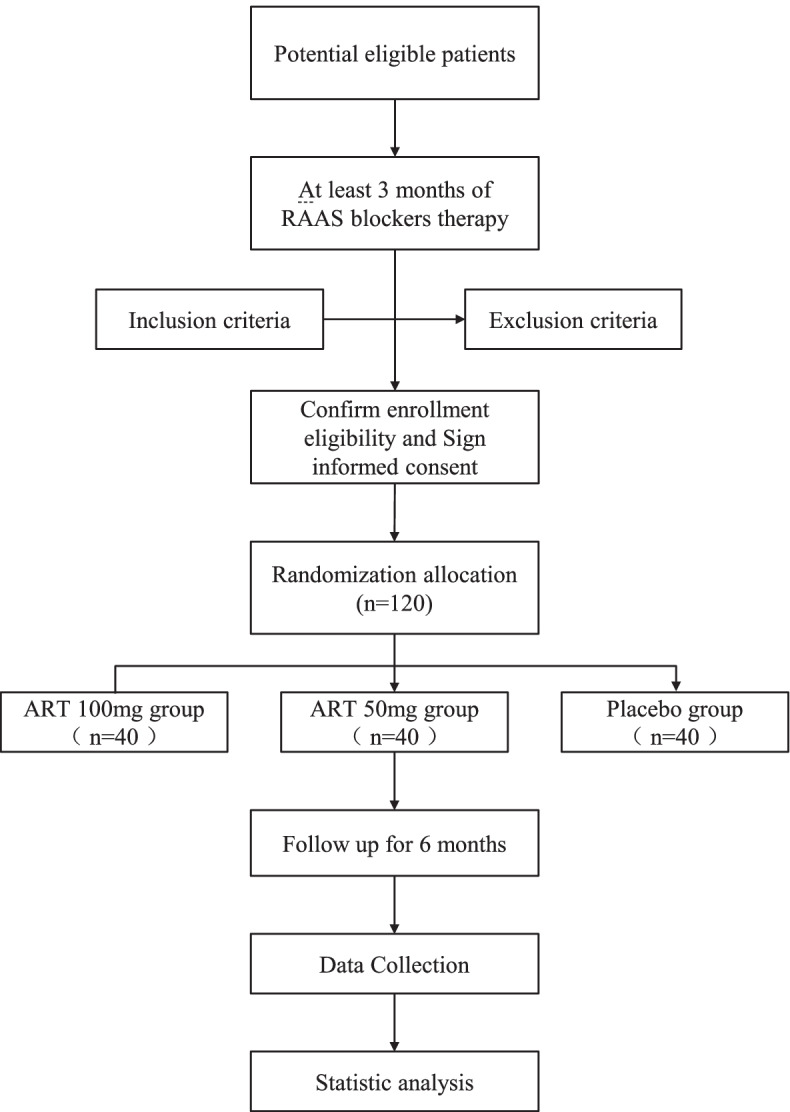
The flow diagram of the study progress

The flow diagram of the study process is shown in Fig. 1. Each participant will be assigned eight visits, including 3 months before the randomization (visit 1), 2 months before the randomization (visit 2), 1 month before the randomization (visit 3), randomization (visit 4), 1 month after the intervention (visit 5), 2 months after the intervention (visit 6), 3 months after the intervention (visit 7), and 6 months after the intervention (visit 8). The schedule for enrollment, intervention, and assessment is shown in Table 1. Retention of patients is promoted by close and frequent contact with their nutritional counselor and study physician via telephone, email, and study visits. In case of relevant issues, visits outside the study schedule are offered.

**Table 1:**
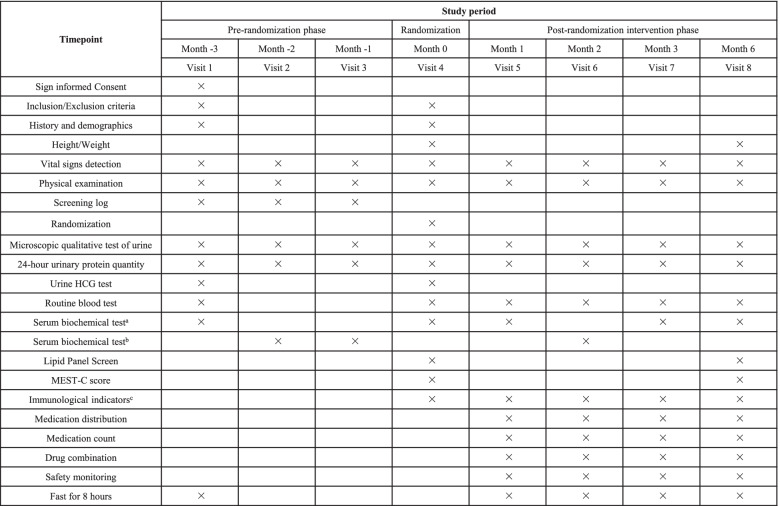
Schedule of recruitment, interventions, and assessments

^a^Items include urea, creatinine, total bilirubin, serum glutamic pyruvic transaminase, serum glutamic-oxaloacetic transaminase, alkaline phosphatase, sodium, potassium, calcium, phosphorus, total protein, albumin, and uric acid

^b^Items include urea, creatinine, sodium, and potassium

^c^Items include Gd-IgA1 and anti-Gd-IgA1(IgG/IgA)

*Abbreviation*: *HCG*, human chorionic gonadotropin

The primary outcome measures will be determined by the percentage change in proteinuria in the three groups after 6 months of intervention. The secondary outcome measures will be the changes in urinary protein creatinine ratio and estimated glomerular filtration rate (eGFR) from baseline after 1, 3, and 6 months of intervention, and the changes in serum galactose-deficient IgA1 (Gd-IgA1) and anti-Gd-IgA1 levels in patients will also be observed. We will stratify the risk of progression of the IgA patients and retrospectively evaluate the effect of ART on the proteinuria levels in IgA patients with different progression risks. Safety assessment of ART will be carried out throughout the study. The study protocol was approved by the Ethics Committee of Dongzhimen Hospital Affiliated with Beijing University of Chinese Medicine (Number: DZMEC-KY-2020-30) and registered in the Chinese Clinical Trial Registry (ChiCTR) (Number: ChiCTR2000038104).

This protocol has been reported according to the Standard Protocol Items: Recommendations for Interventional Trials (SPIRIT) 2013 statement [23]. The SPIRIT checklist is listed in Supplement Table 1. A complete list of clinic hospitals where data will be collected is available in Supplement Table 2.

### Participant recruitment

#### Inclusion criteria

The inclusion criteria for this study are (1) ages 18–75, (2) primary IgA nephropathy confirmed by renal biopsy, (3) persistent proteinuria ≥1.0 g/day after receiving adequate RAAS blocker treatment (proteinuria ≥1.0 g/day at visit 1 and visit 3), and (4) eGFR ≥30 ml/min per 1.73 m^2^ calculated by the Chronic Kidney Disease Epidemiology Collaboration (CKD-EPI) equation.

#### Exclusion criteria

The exclusion criteria included patients who had to be treated with corticosteroid immunotherapy (minimal-change disease with IgA deposition and the presence of crescents in more than 50% of the glomeruli on a renal biopsy within 12 months); patients who must be treated with other immunosuppressants (such as calcineurin inhibitors, cyclophosphamide, or mycophenolate mofetil); patients receiving systemic immunosuppressive therapy in the past 6 months; patients with uncontrolled hypertension (systolic blood pressure >160 mmHg or diastolic blood pressure >110 mmHg); secondary IgA nephropathy; acute kidney injury; malignant tumors; patients with serious diseases of other systems (such as liver damage, heart damage, diseases of the blood system, systemic infections, digestive ulcers); women who are planning to become or are pregnant; and patients with cognitive impairment who cannot sign the informed consent.

#### Withdrawal criteria

The subjects who no longer meet the inclusion criteria, have poor compliance, need immunosuppressive therapy, or have any other reason for violation of the protocol will be monitored by the investigators. These patients will be required to continue regular study visits as scheduled in the protocol after discontinuation of treatment. The data that need to be recorded is the end-point events and safety data that occurred during the duration of this trial.

#### Dropout criteria

Patients who refuse to participate in the study visits for personal reasons or those whom the researchers are unable to contact through all available means will be regarded as dropouts.

#### Termination criteria

Patients will be removed from the study if they develop severe liver or kidney damage during the trial (ALT or AST exceeding twice the normal upper limit, acute kidney injury), severe clinical discomfort caused by the drug (such as severe nausea and vomiting, severe dizziness, and headache), and severe drug allergies. All criteria and decisions will be jointly determined by two or more physicians with the title of deputy chief or higher.

Potential trial participants will be recruited from the six hospitals listed in Supplement Table 2. To reach the target sample size, the trial will be advertised in living communities by local healthcare professionals. We will also utilize advertising via brochures, flyers, or other means suggested through the clinic sites as appropriate. Before each interview, we described the aims of the study, guaranteed confidentiality, and obtained written informed consent. The interviews and examinations were achieved on the premises of the treatment hospital.

### Randomization and allocation

Eligible patients will be randomly divided into the ART 100-mg group, ART 50-mg group, or placebo group at a ratio of 1:1:1. Random numbers will be generated and sorted by independent statisticians using SAS 9.4 (SAS Institute Inc.), and then the patients will be divided into three groups based on the remainder of the number divided by 3. The randomized numbers, rank order, and grouping will be stored in opaque envelopes with serial numbers, which will be kept by the independent researchers and statisticians throughout the study. Envelopes and study drugs will be provided to enroll patients separately by independent blinded researchers.

### Blinding

In this study, a double-blind approach will be applied to the patients and physicians. ART and placebo will be placed in packaged capsules with an identical appearance and distributed to the patients by independent researchers. At the end of the study, independent professional statisticians will be unblinded for the first time and will perform a preliminary analysis of the three sets of outcome data. A second unblinding will be applied to confirm the group and the effect. If serious adverse events occur during the study period, patients will be treated with emergency unblinding and relevant treatment according to the situation.

### Interventions

The intervention will consist of ART 100-mg group oral 50 mg ART tablets twice a day, ART 50-mg group oral 25 mg ART tablets twice a day, and placebo group oral equivalent placebo twice a day. The ART tablets and placebo will be provided by Guilin Nanyao Co., Ltd. All patients will receive standard support treatment for IgA nephropathy, including adequate RAAS blockers and blood pressure control. Permissible combination antihypertensive medications include diuretics, calcium channel blockers, and β-receptor blockers. Hydrochlorothiazide or loop diuretics could be used when antihypertensive targets are difficult to achieve. Statins and aspirins are also allowed when necessary. Prohibited drugs include proprietary Chinese drugs that may reduce proteinuria (such as *Tripterygium wilfordii*, *Penicillium notatum*, Huangkui capsules, nephritis rehabilitation tablets, Haikun Shenxi capsules), corticosteroids, and any immunosuppressants (such as mycophenolate mofetil, cyclophosphamide, azathioprine, HCQ).

### Outcome measurements

The primary outcome is defined as the percentage change of proteinuria in all three groups after 6 months of intervention. Secondary outcomes include the changes in urinary protein creatinine ratio (UPCR), eGFR, and serum Gd-IgA1 levels in all three groups from baseline. The clinical characteristics of the patients will be collected at baseline before enrollment, including age, sex, age, course of the disease, combined disease, medication history, and so on. Throughout the follow-up, patients will be asked to provide urine and blood samples, which will be collected independently at visit 4 and visit 8, which will be collected for the following: collection, storage, and distribution of biological samples and related data for scientific research in the biobank of Dongzhimen hospital. In order to assess drug compliance, participants will be checked for the remaining number of drugs at each follow-up visit. Patient compliance will be calculated as the percentage of the actually taken number of tablets (distributed pills − returned pills) to the distributed number of drugs.

### Safety assessment

The safety assessment includes any serious adverse event and any general adverse event according to the WHO acute and subacute side effects of the performance and indexing criteria for assessment [24]. All records, including adverse events and laboratory test results from the subjects, will be evaluated and followed up until resolved by a medically qualified investigator. The patient’s vital signs and any clinical discomfort will be monitored throughout the study period. Patients who are harmed during this study will receive the necessary medical treatments, such as changing the treatment plan, dealing with complications on time, etc., and the appropriate insurance will cover the expenses.

### Steering committee

The steering committee consists of JW Zhou (chair), JC Lv, LJ Liu, H Li, YH Huo, YB Guo, and C Shen, which is responsible for the design, monitoring, reporting, and publication of the trial. The primary investigator is JW Zhou at Dongzhimen Hospital, sub-investigators are LJ Liu at Peking University First Hospital, H Li at Peking Union Medical College Hospital, YH Huo at the 7th Medical Center of PLA General Hospital, YB Guo at Dongfang Hospital, and C Shen at Beijing Chinese Medicine Hospital. The steering committee will have access to the final trial dataset.

### Data collection and management

Each participant will receive a unique identifier upon study entry. This identifier will be used for all data documentation to ensure the participant’s confidentiality. The entire data collection and preliminary evaluation of the results will be performed by an independent third party. The data will be collected in the form of a case report form (CRF) and then reviewed and stored by the lead researcher in each center. Two researchers will independently convert the original data into electronic storage data to ensure the accuracy of the data. A Data Safety Monitoring Board (DSMB) comprised of independent members, including a statistician and a clinician, has been set up to improve the integrity and credibility of the results. All relevant documentation will be retained so that the safety and key efficacy outcomes can be retrospectively evaluated in the future. No specific interim analysis is foreseen for efficacy unless required by the Steering Committee or DSMB because of safety concerns.

#### Protocol amendments and publications

Important protocol modifications will be communicated to each of the authors and other relevant study investigators and will be updated on ClinicalTrials.gov. IRB approval will be obtained before any important protocol modifications will be implemented. Participants will be notified should any important protocol modifications would concern them. The results from the trial will be submitted for publication in a peer-reviewed journal irrespective of the outcome. The final report will follow the CONSORT 2010 guidelines. Authorship of presentations and reports related to the study will be in the name of the collaborative group.

### Sample size

This is a double-blind, randomized, three-group-parallel, placebo-controlled clinical study, and the sample size estimates were based on the change in proteinuria levels. According to the study results when using similar types of drugs (HCQ) for the treatment of IgA nephropathy [12, 25], the average baseline proteinuria was 2.0±0.8 g/day, and 36 participants in each arm could provide 90% of the detection ability, assuming that the placebo group level of proteinuria had not changed, and the ART treatment detected a 25% reduction in proteinuria (ART group: 0.5±0.5 g/day). Assuming that the two doses of the ART group has the same efficacy (the ratio of the ART group to the placebo group is 2:1), and a 10% dropout rate with a type I error rate of 0.05, we plan to enroll 120 participants (40 in each group) in the study.

### Statistical analysis

All results will be analyzed based on an intention-to-treat (ITT) analysis. Normally distributed data will be expressed as the mean ± standard deviation (SD), while nonnormally distributed data will be expressed as the median of the interquartile interval (IQR). Categorical data will be summarized as counts and percentages. The Shapiro-Wilk test will be performed to determine the normal distribution of continuous variables. For the comparison of baseline characteristics between groups, continuous variables (standard normal distribution) will be analyzed by a one-way ANOVA, continuous variables (nonstandard normal distribution) and ordered categorical variables will be tested by the Kruskal-Wallis *H* test, and unordered categorical variables or count variables will be tested by the chi-square test and Fisher’s exact test.

For the effectiveness outcomes, the primary outcome of proteinuria will be described by the percentage change of 24-h proteinuria test results at each visit from baseline, and the secondary outcomes of UPCR and eGFR will be described by the mean difference between the test results of each visit and the baseline. A paired-samples *t*-test or the Wilcoxon signed-rank test will be used for comparisons within groups at each visit time point from baseline, and one-factor ANOVA and the Kruskal-Wallis *H* test will be used for comparisons among groups at each visit time point.

For the safety indicators, adverse events will be described according to the number of cases and percentage, and the chi-square test and Fisher’s exact test will be used to analyze the differences between groups. The missing values will be filled up by the last-observation-carried-forward method. For the initial analysis, we will not adjust for confounders or stratification, and for the secondary analysis, we will adjust for confounders for subgroup analyses, such as baseline proteinuria (3 g/day), renal function (eGFR 45 m1/min per 1.73 m^2^), blood pressure level (systolic blood pressure 140 mmHg), MEST-C classification [26], and the new international risk-prediction score [27]. SAS 9.4 (SAS Institute Inc.) will be used as the statistical analysis tool. A 2-sided *P* < 0.05 will be defined as statistically significant.

## Discussion

Increasing attention has been given to the application of antimalarial drugs in kidney damage. Given that HCQ has been reported to have a good anti-proteinuria effect in IgA nephropathy patients and that ART has better pharmacological properties than HCQ [28], it is reasonable to believe that ART has the potential to treat IgA nephropathy. Therefore, we designed this double-blind, randomized, three-group-parallel, placebo-controlled clinical trial to evaluate the efficacy and safety of oral ART versus placebo in patients with IgA nephropathy characterized by a high risk of progression in the context of conventional adequate RAAS blocker therapy.

To date, there have been few clinical trials in China on ARSs for the treatment of renal diseases, and only two observational clinical studies on lupus nephritis have reported that ARSs can significantly reduce proteinuria and regulate immune function in patients with lupus nephritis [29, 30]. Studies on the treatment of IgA nephropathy with ART have been mainly based on animal models and cell experiments, and a large amount of laboratory evidence has shown that the effect of ART on IgA nephropathy may be primarily due to its strong anti-inflammatory and immune-regulatory properties, as well as its ability to regulate oxidative stress [31]. Animal experiments found that the combination of ARS and HCQ can reduce the levels of 24-h urine protein, IgA, and IgG immune complex deposition in the glomerulus through NF-κB/NLRP3 signaling [22] and improve the barrier function of the glomerular filtration membrane by regulating the immune response in IgA nephropathy rats [32]. One team demonstrated that dihydroartemisinin (the main active metabolite of ART) could attenuate the proliferation of human mesangial cells induced by aggregated IgA1 through the mTOR signaling pathway in vitro [33]. Although there is a lot of current laboratory evidence that ART has a good regulatory effect on IgA nephropathy, there are still no high-quality randomized, placebo-controlled clinical trials to provide evidence of ART in the treatment of IgA nephropathy, which has limited the clinical use of ART in IgA nephropathy.

IgA nephropathy is widely accepted to be a consequence of four hits: circulating IgA1 bearing galactose-deficient O-glycans (Gd-IgA1) in patients with IgA nephropathy is increased (hit 1), and these IgA1 glycobodies are recognized as autoantigens by antiglycan autoantibodies (anti-Gd-IgA1 autoantibodies; hit 2), which leads to the formation of an immune complex in nephritis (hit 3), some of which is deposited in the kidney and activates mesangial cells (hit 4), resulting in kidney injury [34]. The Gd-IgA1 molecule, which is a typical feature of IgAN, is produced by IgA1-secreting cells (normal functioning B cells) through abnormal biosynthesis of O-glycans [35]. When B cells are programmed to sialylate IgA1 in the early stage of posttranslational glycosylation (the addition of galactose is excluded), the formation of IgA1-containing immune complexes in IgAN can be changed [36]. The accumulated evidence shows that the possible mechanism of ART in treating IgA nephropathy includes two aspects: ARS can inhibit the production of inflammatory cytokines (such as IL-6 and TGF-β) and block the overactivation of B lymphocytes [37], inhibit the synthesis of Gd-IgA1, and reduce the production of IgG and/or IgA1 autoantibodies, reducing the severity of IgA nephropathy. ARS can also reduce the inflammatory cascade caused by mesangial cell activation by inhibiting the production of cytokines [38] (such as the NF-κB signal transduction pathway) and alleviate the subsequent damage to podocytes and tubules, thus reducing proteinuria.

Our study benefits from our larger sample size and the strict design as a multicenter, three-group-parallel study with placebo controls, and we will observe the effects of ART on the serum Gd-IgA levels in patients with IgA nephropathy, which will help us better understand the proteinuria reduction effect of ART and its possible mechanism. The limitation of this study is that it is a proof-of-concept study and will only follow the patients up for 6 months, which may not initially provide clear answers to more mechanistic questions. If this study achieves the expected results at the end of the trial, additional clinical studies with longer follow-up periods will be conducted to confirm whether ART could be an alternative treatment option for IgA nephropathy in the future.

## Trial status

This is version 2.0, 23 April 2020. Recruitment will start on 1 July 2021, and we anticipate that the trial will finish recruitment on 31 December 2022.

## Supplementary Information

**Additional file 1.** : SPIRIT 2013 checklist.

**Additional file 2.** : A complete list of clinic hospitals where data will be collected.

## Data Availability

The datasets used and/or analyzed during the current study are available from the corresponding author on reasonable request and appropriate human subject approval.

## Abbreviations

IgA: Immunoglobulin A
ESRD: End-stage renal disease
HCQ: Hydroxychloroquine
ART: Artesunate
ARS: Artemisinin
CQ: Chloroquine
SPIRIT: The Standard Protocol Items: Recommendations for Interventional Trials
UPCR: Urinary protein creatinine ratio
ITT: Intention-to-treat
SD: Standard deviation
IQR: Interquartile interval
