# Supplementary material for: Efficacy and safety of artesunate for patients with IgA nephropathy: a study protocol for a multicenter, double-blind, randomized, placebo-controlled trial
Source: Trials. 2022 May 25;23:444. doi: 10.1186/s13063-022-06336-3 (PMC9134594; doi:10.1186/s13063-022-06336-3)
Supplement: Supplementary file 1 — Additional file 1. : SPIRIT 2013 checklist. [file 13063_2022_6336_MOESM1_ESM.pdf]

SPIRIT 2013 Checklist: Recommended items to address in a clinical trial protocol and related documents*

Section/item	Item No	Description	Reported on Page Number/Line Number	Reported on Section/Paragraph
**Administrative information**				
Title	1	Descriptive title identifying the study design, population, interventions, and, if applicable, trial acronym	Page 1/Line 1-2	Title
Trial registration	2a	Trial identifier and registry name. If not yet registered, name of intended registry	Page 2/Line 12	Methods and Design
	2b	All items from the World Health Organization Trial Registration Data Set	Page 6/Line 2	Abstract
Protocol version	3	Date and version identifier	Page 17/Line 15	Trial status
Funding	4	Sources and types of financial, material, and other support	Page 18/Line 11	Funding
Roles and responsibilities	5a	Names, affiliations, and roles of protocol contributors	Page 18/Line 17	Authors' contributions
	5b	Name and contact information for the trial sponsor	Page 18/Line 11	Funding
	5c	Role of study sponsor and funders, if any, in study design; collection, management, analysis, and interpretation of data; writing of the report; and the decision to submit the report for publication, including whether they will have ultimate authority over any of these activities	Page 18/Line 11	Funding
	5d	Composition, roles, and responsibilities of the coordinating centre, steering committee, endpoint adjudication committee, data management team, and other individuals or groups overseeing the trial, if applicable (see Item 21a for data monitoring committee)	Page 12/Line 4	Methods and Design
**Introduction**				
Background and rationale	6a	Description of research question and justification for undertaking the trial, including summary of relevant studies (published and unpublished) examining benefits and harms for each intervention	Page 3/Line 3	Introduction
	6b	Explanation for choice of comparators	Page 3/Line 115	Introduction
Objectives	7	Specific objectives or hypotheses	Page 5/Line 17	Introduction

Trial design	8	Description of trial design including type of trial (eg, parallel group, crossover, factorial, single group), allocation ratio, and framework (eg, superiority, equivalence, noninferiority, exploratory)	Page 6/Line 6	Methods and Design
**Methods: Participants, interventions, and outcomes**				
Study setting	9	Description of study settings (eg, community clinic, academic hospital) and list of countries where data will be collected. Reference to where list of study sites can be obtained	Page 7Line 17	Methods and Design
Eligibility criteria	10	Inclusion and exclusion criteria for participants. If applicable, eligibility criteria for study centres and individuals who will perform the interventions (eg, surgeons, psychotherapists)	Page 7/Line 20	Methods and Design
Interventions	11a	Interventions for each group with sufficient detail to allow replication, including how and when they will be administered	Page 10/Line 14	Methods and Design
	11b	Criteria for discontinuing or modifying allocated interventions for a given trial participant (eg, drug dose change in response to harms, participant request, or improving/worsening disease)	Page 8/Line 16	Methods and Design
	11c	Strategies to improve adherence to intervention protocols, and any procedures for monitoring adherence (eg, drug tablet return, laboratory tests)	Page 9/Line 11	Methods and Design
	11d	Relevant concomitant care and interventions that are permitted or prohibited during the trial	Page 10/Line 22	Methods and Design
Outcomes	12	Primary, secondary, and other outcomes, including the specific measurement variable (eg, systolic blood pressure), analysis metric (eg, change from baseline, final value, time to event), method of aggregation (eg, median, proportion), and time point for each outcome. Explanation of the clinical relevance of chosen efficacy and harm outcomes is strongly recommended	Page 11/Line 4	Methods and Design
Participant timeline	13	Time schedule of enrolment, interventions (including any run-ins and washouts), assessments, and visits for participants. A schematic diagram is highly recommended (see Figure)	Page 6/Line 17	Methods and Design
Sample size	14	Estimated number of participants needed to achieve study objectives and how it was determined, including clinical and statistical assumptions supporting any sample size calculations	Page 13/Line 12	Methods and Design
Recruitment	15	Strategies for achieving adequate participant enrolment to reach target sample size	Page 10/Line 22	Methods and Design
**Methods: Assignment of interventions (for controlled trials)**				
Allocation:				
Sequence generation	16a	Method of generating the allocation sequence (eg, computer-generated random numbers), and list of any factors for stratification. To reduce predictability of a random sequence, details of any planned restriction (eg, blocking) should be provided in a separate document that is unavailable to those who enrol participants or assign interventions	Page 9/Line 19	Methods and Design
Allocation concealment mechanism	16b	Mechanism of implementing the allocation sequence (eg, central telephone; sequentially numbered, opaque, sealed envelopes), describing any steps to conceal the sequence until interventions are assigned	Page 9/Line 19	Methods and Design

Implementation	16c	Who will generate the allocation sequence, who will enrol participants, and who will assign participants to interventions	Page 9/Line 19	Methods and Design
Blinding (masking)	17a	Who will be blinded after assignment to interventions (eg, trial participants, care providers, outcome assessors, data analysts), and how	Page 10/Line 5	Methods and Design
	17b	If blinded, circumstances under which unblinding is permissible, and procedure for revealing a participant's allocated intervention during the trial	Page 10/Line 5	Methods and Design
**Methods: Data collection, management, and analysis**				
Data collection methods	18a	Plans for assessment and collection of outcome, baseline, and other trial data, including any related processes to promote data quality (eg, duplicate measurements, training of assessors) and a description of study instruments (eg, questionnaires, laboratory tests) along with their reliability and validity, if known. Reference to where data collection forms can be found, if not in the protocol	Page 12/Line 12	Methods and Design
	18b	Plans to promote participant retention and complete follow-up, including list of any outcome data to be collected for participants who discontinue or deviate from intervention protocols	Page 12/Line 12	Methods and Design
Data management	19	Plans for data entry, coding, security, and storage, including any related processes to promote data quality (eg, double data entry; range checks for data values). Reference to where details of data management procedures can be found, if not in the protocol	Page 12/Line 12	Methods and Design
Statistical methods	20a	Statistical methods for analysing primary and secondary outcomes. Reference to where other details of the statistical analysis plan can be found, if not in the protocol	Page 13/Line 23	Methods and Design
	20b	Methods for any additional analyses (eg, subgroup and adjusted analyses)	Page 13/Line 23	Methods and Design
	20c	Definition of analysis population relating to protocol non-adherence (eg, as randomised analysis), and any statistical methods to handle missing data (eg, multiple imputation)	Page 13/Line 23	Methods and Design
**Methods: Monitoring**				
Data monitoring	21a	Composition of data monitoring committee (DMC); summary of its role and reporting structure; statement of whether it is independent from the sponsor and competing interests; and reference to where further details about its charter can be found, if not in the protocol. Alternatively, an explanation of why a DMC is not needed	Page 12/Line 19	Methods and Design
	21b	Description of any interim analyses and stopping guidelines, including who will have access to these interim results and make the final decision to terminate the trial	Page 12/Line 22	Methods and Design
Harms	22	Plans for collecting, assessing, reporting, and managing solicited and spontaneously reported adverse events and other unintended effects of trial interventions or trial conduct	Page 12/Line 1	Methods and Design
Auditing	23	Frequency and procedures for auditing trial conduct, if any, and whether the process will be independent from investigators and the sponsor	Page 11/Line 10	Methods and Design

**Ethics and dissemination**				
Research ethics approval	24	Plans for seeking research ethics committee/institutional review board (REC/IRB) approval	Page 7/Line 11	Methods and Design
Protocol amendments	25	Plans for communicating important protocol modifications (eg, changes to eligibility criteria, outcomes, analyses) to relevant parties (eg, investigators, REC/IRBs, trial participants, trial registries, journals, regulators)	Page 13/Line 3	Methods and Design
Consent or assent	26a	Who will obtain informed consent or assent from potential trial participants or authorised surrogates, and how (see Item 32)	Page 9/Line 16	Methods and Design
	26b	Additional consent provisions for collection and use of participant data and biological specimens in ancillary studies, if applicable	Supplement	Methods and Design
Confidentiality	27	How personal information about potential and enrolled participants will be collected, shared, and maintained in order to protect confidentiality before, during, and after the trial	Page 12/Line 13	Methods and Design
Declaration of interests	28	Financial and other competing interests for principal investigators for the overall trial and each study site	Page 18/Line 11	Declarations
Access to data	29	Statement of who will have access to the final trial dataset, and disclosure of contractual agreements that limit such access for investigators	Page 18/Line 6	Declarations
Ancillary and post-trial care	30	Provisions, if any, for ancillary and post-trial care, and for compensation to those who suffer harm from trial participation	Page 12/Line 1	Methods and Design
Dissemination policy	31a	Plans for investigators and sponsor to communicate trial results to participants, healthcare professionals, the public, and other relevant groups (eg, via publication, reporting in results databases, or other data sharing arrangements), including any publication restrictions	Page 13/Line 3	Methods and Design
	31b	Authorship eligibility guidelines and any intended use of professional writers	Page 18/Line 16	Declarations
	31c	Plans, if any, for granting public access to the full protocol, participant-level dataset, and statistical code	Page 13/Line 18	Declarations
**Appendices**				
Informed consent materials	32	Model consent form and other related documentation given to participants and authorised surrogates	Supplement	Supplement
Biological specimens	33	Plans for collection, laboratory evaluation, and storage of biological specimens for genetic or molecular analysis in the current trial and for future use in ancillary studies, if applicable	Supplement	Supplement

*It is strongly recommended that this checklist be read in conjunction with the SPIRIT 2013 Explanation & Elaboration for important clarification on the items. Amendments to the protocol should be tracked and dated. The SPIRIT checklist is copyrighted by the SPIRIT Group under the Creative Commons "Attribution-NonCommercial-NoDerivs 3.0 Unported" license.

Please leave this space alone as it will be supplemented by the editorial office when needed.
