# Supplementary material for: Efficacy and safety of artesunate for patients with IgA nephropathy: a study protocol for a multicenter, double-blind, randomized, placebo-controlled trial
Source: Trials. 2022 May 25;23:444. doi: 10.1186/s13063-022-06336-3 (PMC9134594; doi:10.1186/s13063-022-06336-3)
Supplement: Supplementary file 2 — Additional file 2. : A complete list of clinic hospitals where data will be collected. [file 13063_2022_6336_MOESM2_ESM.pdf]

List of Studay Centres

Centres	Department office	Investigator	Degree	Email	Telephone	Role	Address
Peking University First Hospital	Department of Nephrology	Lijun Liu	Doctor of Medicine	Lijun.liu@aliyun.com	010-83572211	Sub-center	Beijing, 100007, China
Peking University First Hospital	Department of Nephrology	Hang Li	Doctor of Medicine	lihang9 @hotmail.com	010-65295056	Sub-center	Beijing, 100007, China
Dongzhimen Hospital	Department of Nephrology	Jingwei Zhou	Doctor of Medicine	13910634708@163.com	010-84013142	Principal center	Beijing, 100007, China
the 7th Medical Center of PLA General Hospital	Department of Nephrology	Yanhong Huo	Doctor of Medicine	13691110882@139.com	010-66721629	Sub-center	Beijing, 100007, China
Dongfang Hospital	Department of Nephrology	Yingbo Guo	Doctor of Medicine	guoyingbo0603@sina.com	010-67689974	Sub-center	Beijing, 100007, China
Beijing Chinese Medicine Hospital Affiliated to Capital Medical University	Department of Nephrology	Cun Shen	Doctor of Medicine	shencun0228@163.com	010-52176631	Sub-center	Beijing, 100007, China
